# Measuring Risky Driving Behavior Using an mHealth Smartphone App: Development and Evaluation of gForce

**DOI:** 10.2196/mhealth.9290

**Published:** 2018-04-19

**Authors:** Raisa Z Freidlin, Amisha D Dave, Benjamin G Espey, Sean T Stanley, Marcial A Garmendia, Randall Pursley, Johnathon P Ehsani, Bruce G Simons-Morton, Thomas J Pohida

**Affiliations:** ^1^ Center for Information Technology National Institutes of Health Bethesda, MD United States; ^2^ Biomedical Engineering Department University of Connecticut Storrs, CT United States; ^3^ Bloomberg School of Public Health Johns Hopkins University Baltimore, MD United States; ^4^ National Institute of Child Health and Human Development National Institutes of Health Bethesda, MD United States

**Keywords:** kinematic risky driving behavior, naturalistic driving studies, elevated g-force, lateral acceleration, longitudinal acceleration, iPhone

## Abstract

**Background:**

Naturalistic driving studies, designed to objectively assess driving behavior and outcomes, are conducted by equipping vehicles with dedicated instrumentation (eg, accelerometers, gyroscopes, Global Positioning System, and cameras) that provide continuous recording of acceleration, location, videos, and still images for eventual retrieval and analyses. However, this research is limited by several factors: the cost of equipment installation; management and storage of the large amounts of data collected; and data reduction, coding, and analyses. Modern smartphone technology includes accelerometers built into phones, and the vast, global proliferation of smartphones could provide a possible low-cost alternative for assessing kinematic risky driving.

**Objective:**

We evaluated an in-house developed iPhone app (gForce) for detecting elevated g-force events by comparing the iPhone linear acceleration measurements with corresponding acceleration measurements obtained with both a custom Android app and the in-vehicle miniDAS data acquisition system (DAS; Virginia Tech Transportation Institute).

**Methods:**

The iPhone and Android devices were dashboard-mounted in a vehicle equipped with the DAS instrumentation. The experimental protocol consisted of driving maneuvers on a test track, such as cornering, braking, and turning that were performed at different acceleration levels (ie, mild, moderate, or hard). The iPhone gForce app recorded linear acceleration (ie, gravity-corrected). The Android app recorded gravity-corrected and uncorrected acceleration measurements, and the DAS device recorded gravity-uncorrected acceleration measurements. Lateral and longitudinal acceleration measures were compared.

**Results:**

The correlation coefficients between the iPhone and DAS acceleration measurements were slightly lower compared to the correlation coefficients between the Android and DAS, possibly due to the gravity correction on the iPhone. Averaging the correlation coefficients for all maneuvers, the longitudinal and lateral acceleration measurements between iPhone and DAS were *r*_lng_=0.71 and *r*_lat_=0.83, respectively, while the corresponding acceleration measurements between Android and DAS were *r*_lng_=0.95 and *r*_lat_=0.97. The correlation coefficients between lateral accelerations on all three devices were higher than with the corresponding longitudinal accelerations for most maneuvers.

**Conclusions:**

The gForce iPhone app reliably assessed elevated g-force events compared to the DAS. Collectively, the gForce app and iPhone platform have the potential to serve as feature-rich, inexpensive, scalable, and open-source tool for assessment of kinematic risky driving events, with potential for research and feedback forms of intervention.

## Introduction

Teenage drivers, compared to other age groups, have the highest risk of fatal automobile crashes per driven mile [1]. In 2015, there were 221,313 teenage drivers admitted to hospital emergency rooms, which resulted in 2333 fatalities (Centers for Disease Control statistics, average of 6 per day) [2]. Contributing factors to teenage crashes include night-time driving, speeding, impairment due to alcohol or other drugs, and secondary task engagement such as mobile phone use, teenage passenger presence, and eating [3,4].

While there is substantial individual variability in risk [5] and rapid improvement in driving outcomes with age and experience, the first year of driving is particularly risky [6,7]. The high risk among novice teenage drivers has been attributed to young age, inexperience, and risky driving behaviors [7]. A unique characteristic of teenage risky driving is a high rate of elevated acceleration (g-force) events due to hard stops, rapid starts, sharp turns, and over-correction maneuvers, reflecting poor speed control or volitionally erratic driving [8]. The rate of elevated g-force events, termed kinematic risky driving (KRD), are 4-5 times higher among young drivers than adults [8] and are prospectively associated with crash likelihood [9]. KRD can be assessed by accelerometers installed in vehicles in naturalistic driving studies (NDSs) and are sometimes employed to provide driving performance feedback to teenage drivers and their parents [10]. NDSs [11,12], designed to objectively assess driving behavior and outcomes, are conducted by equipping vehicles with dedicated instrumentation (eg, accelerometers, gyroscopes, Global Positioning System [GPS], and cameras) that provide continuous recording of acceleration, location, videos, and still images for eventual retrieval and analyses. However, this research is limited by several factors: the cost of equipment installation; management and storage of the large amounts of data collected; and data reduction, coding, and analyses. Modern smartphone technology includes accelerometers built into phones, and the vast, global proliferation of smartphones could provide a possible low-cost alternative for assessing KRD.

Research to date on driver-monitoring smartphone apps have mainly focused on monitoring driver behavior as indicated by vehicle lateral and longitudinal accelerometer data [13-16]. Unfortunately, most of these apps do not provide features (eg, video capture, event selectivity, real-time feedback) or tools (eg, downstream data collection, processing, and reporting) for a comprehensive analysis of events. Currently, smartphone video capability has only been used for driver fatigue detection, disregarding other driver distractions such as eating or drinking. Applying these existing apps to driving research presents significant challenges, including the following: (1) proprietary code (eg, not open source, therefore not modifiable); and (2) data accessibility, scalability, and research suitability (eg, provide raw data) for innovative data analysis and development of methods to provide feedback as a means of preventive intervention.

Advances in mHealth solutions and data-centralization methods, including smartphone assessments, could result in reduced cost and complexity associated with collecting comprehensive driving data, and enabling increased and innovative driving research. Additionally, driving data could be collected by smartphones from previously unmeasured populations (eg, lower socio-economic strata, low-income countries), which could result in further app diversification, research insights, and a wider impact on driving safety.

A recent survey confirmed the continued popularity and dominant market share of the iPhone among teenagers; 81% of surveyed US teenagers owned an iPhone, and this number is projected to grow [17]. However, no research has reported the utility of the iPhone for assessing KRD. The purpose of this work was to evaluate the utility of a simple, nonproprietary iPhone app to assess teenage KRD behavior. The research evaluates the feasibility of using iPhone devices as a research tool for NDS by comparing linear acceleration acquired with iPhone devices to acceleration measurements obtained simultaneously with an Android smartphone (Samsung Galaxy S5) and the in-vehicle miniDAS data acquisition system (DAS) developed at the Virginia Tech Transportation Institute (VTTI).

## Methods

### Overview

The smartphone Application to Measure Risky Driving Behavior and Predict Crashes (gForce App), was developed in Swift, which is a general-purpose, multi-platform, programming language for iOS using an Xcode environment (Apple, Inc). The gForce App was tested on the iPhone 6 (Apple, Inc) running iOS 10.3.1. The iPhone incorporates the Sensortec BMA280 3-axis accelerometer and the InvenSense MP67B 6-axis accelerometer, gyroscope, and magnetometer combined sensor with an on-chip digital motion processor (DMP) with sensor-fusion capabilities. Sensor-fusion is a technology (ie, firmware) that algorithmically combines data from multiple sensors to mitigate the limitations of the individual sensors to more accurately calculate the real-time position and orientation of the iPhone device. In addition, but not part of this research, the gForce app utilizes the iPhone dual cameras (ie, front and back cameras). The front camera records a video of the driver, while the back camera captures still images outside the vehicle.

The gForce App is designed to continuously record (ie, 10 Hz nominal sampling rate) linear acceleration (acceleration of the device, excluding the effect of gravity on the device) and rotation data for the x, y, and z axes. Immediately, the gForce App calculates a directionless g-force by combining the linear acceleration data from all three axes. The data is stamped with Coordinated Universal Time and GPS location. Other app features include: an audio warning when a g-force event is triggered, a capability of uploading JSON formatted files to a centralized database, and a fully integrated navigation solution.

This navigation feature may reduce the driver’s desire to switch to another app, which, due to iOS limitations, would effectively disable gForce camera acquisitions even though the app would continue to run otherwise (eg, measure and upload g-forces).

The iPhone acceleration measurements were compared with the acceleration values acquired by a custom-built app on a Galaxy S5 (Samsung, Inc) running Android 4.5.2 (Google, Inc), and the DAS. Similar to the iPhone gForce App, the Android app and DAS system continuously record acceleration and rotation measurements along three axes, stamped with time and location. The Galaxy S5 utilizes a InvenSense MP65M 6-axis gyroscope, accelerometer, and magnetometer-combined sensor with a DMP. The DAS device is equipped with a STMicroelectronics LSM303DLM sensor module, which includes a 3-axis accelerometer and three-axis magnetometer. The DAS device does not provide onboard sensor-fusion technology. Similar to the iPhone gForce App, the Android app features wireless uploading of data to a centralized database in JSON and, in addition, CSV file format. The DAS system requires data transfer via a Secure Digital card. The Android app was pilot tested and refined based in small-scale field tests in Washington D.C. and was compared to the DAS in a previous experiment [18]. The DAS has been widely employed in NDSs and is considered to provide reliable g-force measures [9,12].

### Procedures

Device testing consisted of the following two parts: (1) test track driving on the Virginia Smart Road research facility, which is managed by VTTI and owned and maintained by the Virginia Department of Transportation; and (2) street driving in Christiansburg, VA.

**Figure 1:**
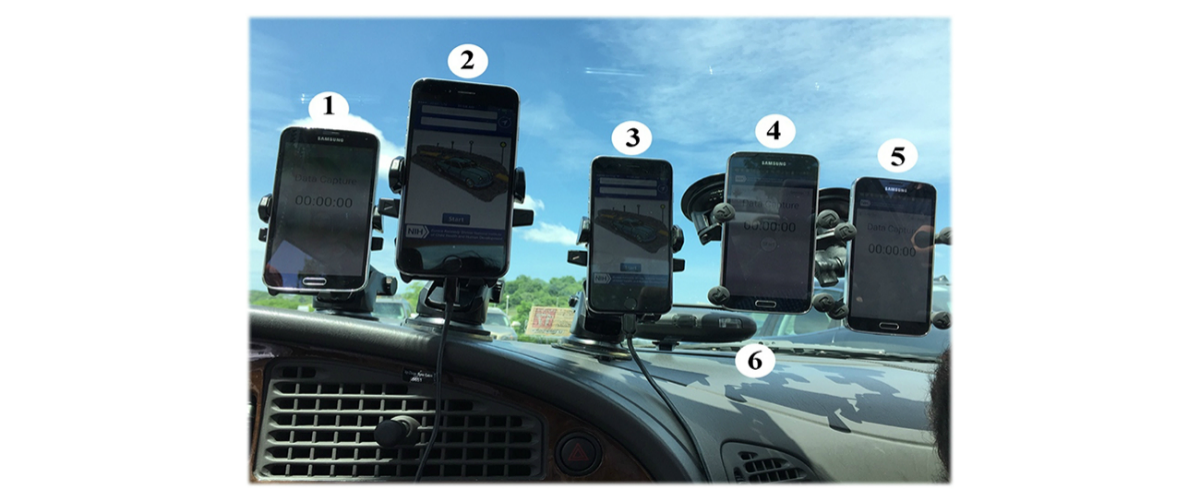
Smartphones used for the Virginia Tech Transportation Institute road test: iPhone devices are #2 and 3; Android devices are #1, 4, and 5; Virginia Tech Transportation Institute permanently vehicle-installed instrumentation #6.

**Figure 2:**
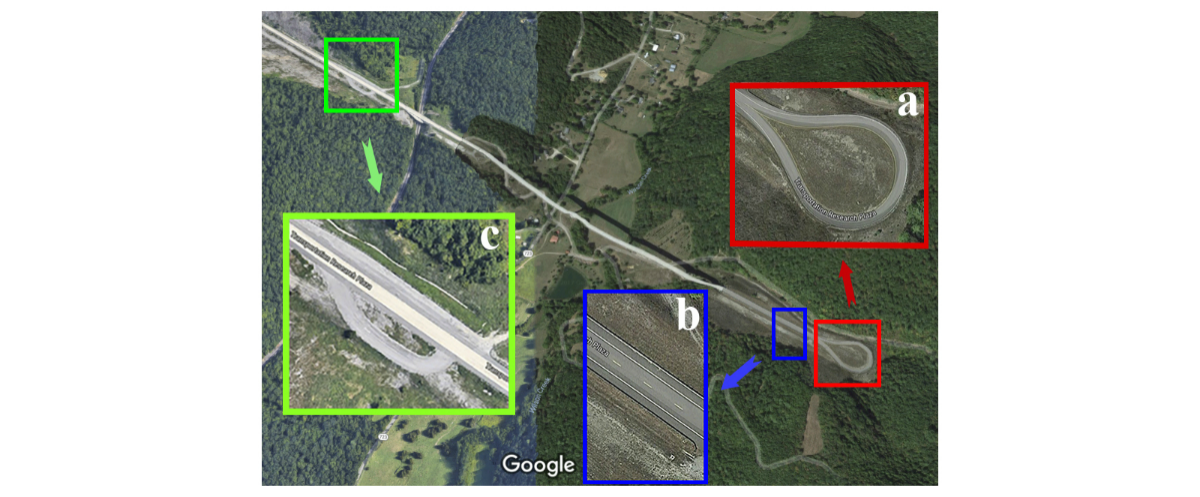
Virginia Smart Road: a) roundabout used for cornering; b) straight portion of the road used for braking and acceleration; c) turnout lane used for turning left and right.

**Figure 3:**
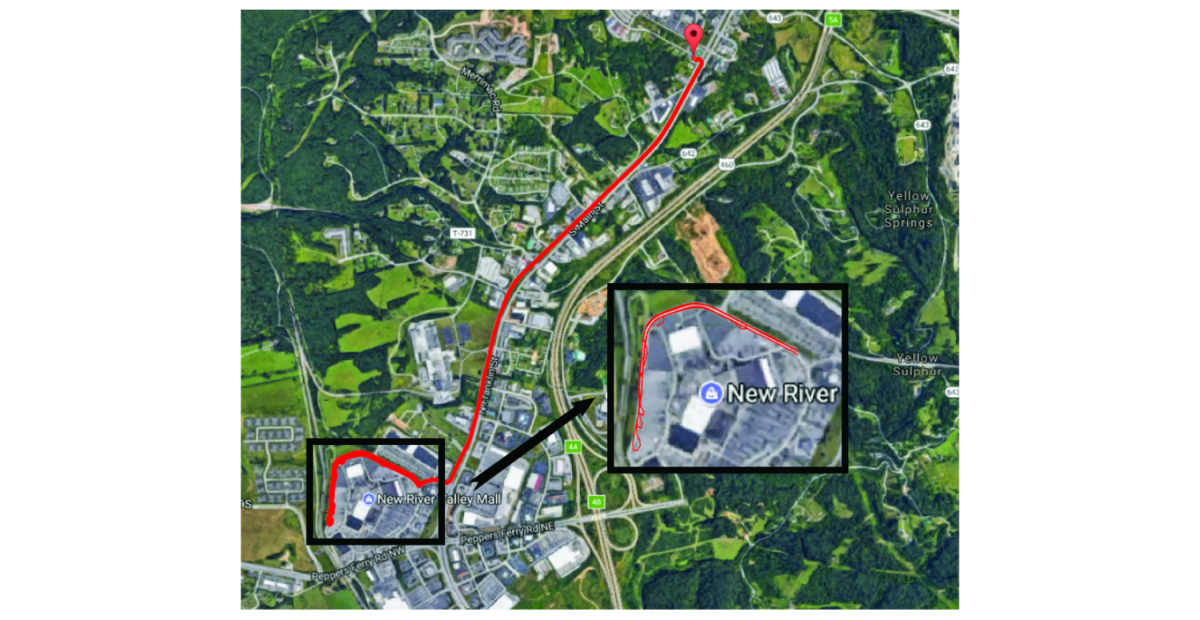
The street-driving phase of the test in Christiansburg, VA. Driving over speedbumps, potholes and U-turns was performed on the loop around New River Valley Mall (black box).

### Experimental Protocol

Figure 1 shows the orientation and location of the devices used in this study. These devices included an Android phone (#1) and two iPhones (#2 and #3) mounted on the dashboard, two Android phones (#4 and #5) mounted on the windshield, and the permanently installed DAS device (#6). A fourth Android phone (not shown) provided the driver with a g-force estimation during each maneuver, which helped to ensure the desired g-forces were generated. Experimental testing on the Smart Road consisted of consecutive groups of driving maneuvers, including: 10 moderate and 5 hard cornering for each left and right directions on a roundabout (Figure 2 a), braking (15 hard) and acceleration (5 mild) driving in a straight line (Figure 2 b), and 12 moderate and 6 hard turning maneuvers in each left and right directions (Figure 2 c). The street-driving phase of the test (Christiansburg, VA, Figure 3) was comprised of normal driving maneuvers and road conditions, such as: 3 stop signs, 2 traffic lights, 2 left and 6 right turns, 4 left and 2 right U-turns, 7 left and 5 right cornering, 14 speedbumps, and 8 potholes. The duration of the test was 10 minutes 14 seconds. There were four passengers in the car.

### Data Processing and Analysis

Postprocessing was performed in MATLAB (MathWorks, Inc). To reduce noise and the number of false positives (ie, g-force events associated with speedbumps and potholes), a second-order low-pass Butterworth digital filter with a cutoff frequency of 0.4 Hz was applied to iPhone and Android measurements. DAS sensor data was processed on the device with a 5 Hz low-pass filter, followed by a MATLAB low-pass filter with parameters identical to iPhone and Android data post-processing. The Pearson correlation coefficients, *r*, for acceleration along x (lateral, *r*_lat_), y, and z (longitudinal, *r*_lng_) axes were estimated for the iPhone (the average of two iPhones) versus DAS, and Android (the average of two Androids) versus DAS.

Lateral (ie, x-axis) acceleration corresponds to sideways movement in relationship to the direction of travel, while longitudinal (ie, z-axis) acceleration corresponds to acceleration in the direction of travel. In this work, we report correlation coefficients for the longitudinal and lateral acceleration measurements only. The average duration of the g-force events over 0.45 g were estimated for each device. This threshold was selected for all events to provide consistent values for analyses.

## Results

### Gravity Correction on the Android and Data Acquisition System

**Figure 4:**
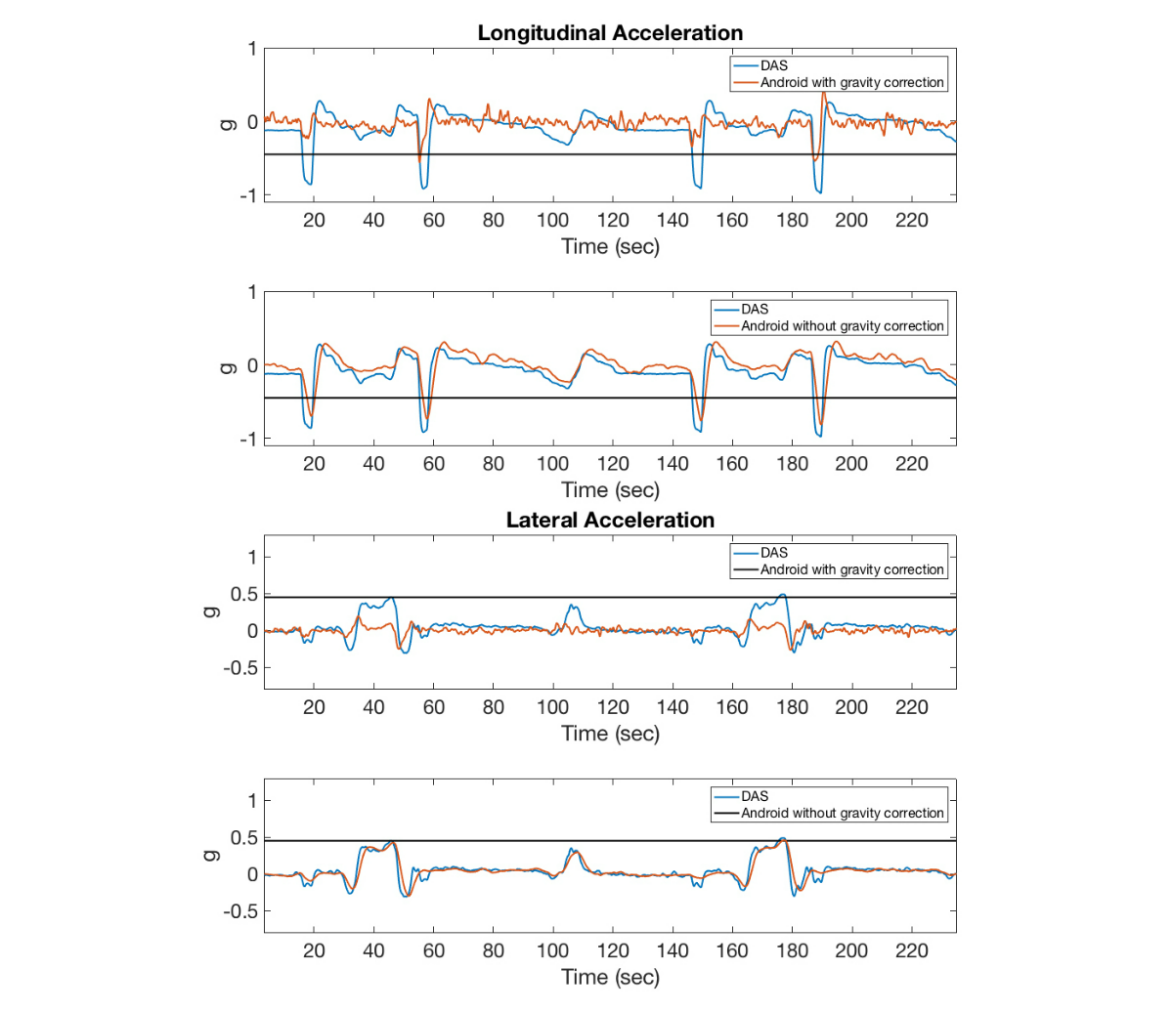
Comparison of gravity-corrected and uncorrected Android acceleration measurements with data acquisition system (DAS; gravity-uncorrected).

**Figure 5:**
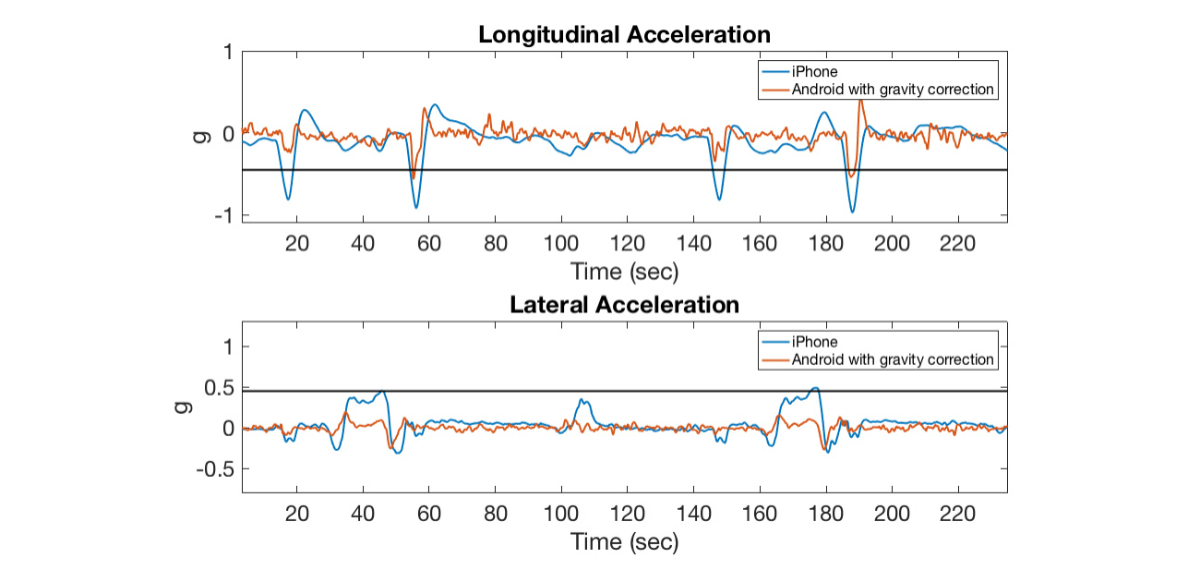
Comparison of gravity-corrected Android acceleration measurements with iPhone (inherently gravity-corrected).

Preliminary analyses indicated a poor correlation between the Android linear acceleration and the DAS acceleration measurements. In addition, the amplitude of the signal consistently below the threshold of 0.45 g for all maneuvers revealed that the gravity correction on the Android device was ineffective in correctly adjusting for the effect of gravity. This gravity correction issue was especially pronounced during the braking maneuvers where the correlation coefficients between the Android linear longitudinal (Figure 4 a) and lateral (Figure 4 b) acceleration and DAS acceleration measurements were *r*_lng_=0.05 and *r*_lat_=0.10, respectively, while the correlation coefficients for the same maneuvers without gravity correction on the Android device were *r*_lng_=0.93 and *r*_lat_=0.89 for the longitudinal and lateral acceleration measurements, respectively. Figure 5 shows a similar trend between the Android and iPhone gravity-corrected acceleration measurements. Therefore, in this work, the Android acceleration measurements without gravity correction were used for estimating correlation coefficients between the Android and DAS devices.

### Effect of Weight Distribution on Kinematic Measures

**Figure 6:**
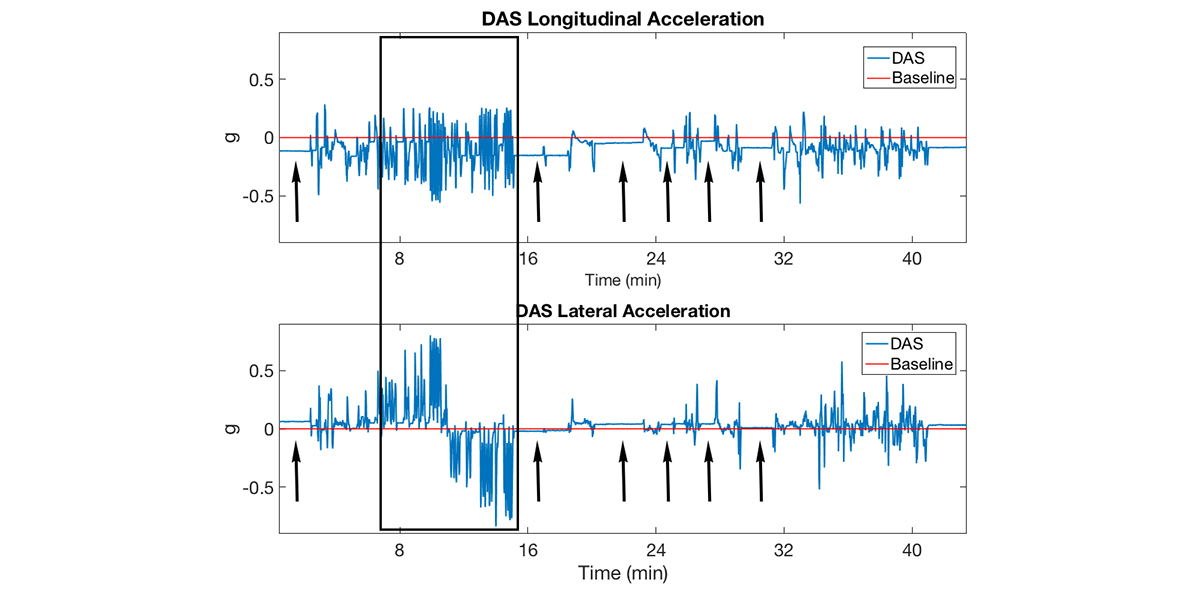
The effects of in-vehicle weight distribution on baseline acceleration measurements. The acceleration measurements offset from the baseline varied (arrows) based on the number of passengers/weight distribution inside the vehicle. Comparison with the Android and iPhone devices within the black box is show in Figure 7. DAS: data acquisition system.

**Figure 7:**
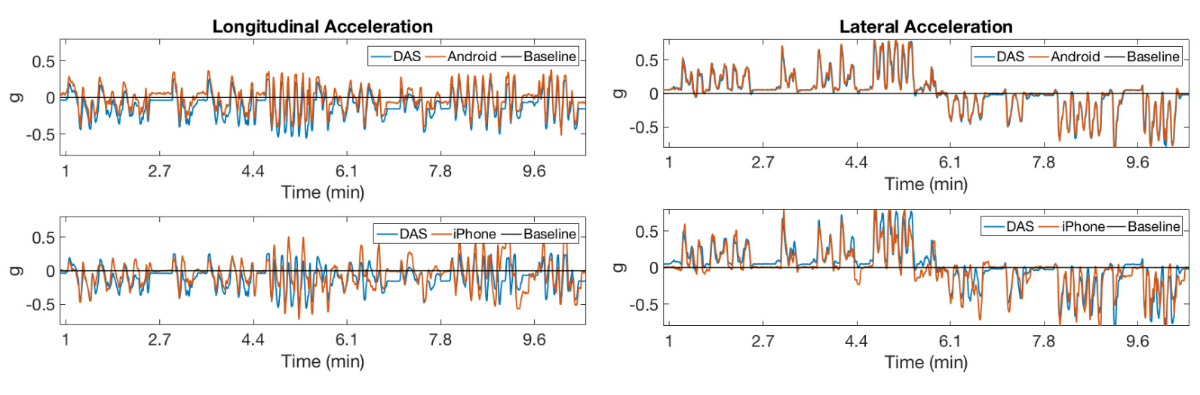
The effects of in-vehicle weight distribution on baseline acceleration measurements. While vehicle is stationary, the acceleration measurements should be zero. DAS: data acquisition system.

The weight distribution (ie, vehicle lean) within the vehicle (ie, number of passengers and their location) had an effect on the DAS acceleration measurements. As seen in Figure 6, the baseline offset shifted (black arrows) as passengers entered or exited the vehicle. Figure 7 shows the Android acceleration measurements were similarly affected by weight distribution, while the iPhone baseline offset correctly remained at zero.

Figure 6 and Figure 7 show that in the absence of gravity correction, the DAS and Android acceleration amplitude measurements were either overestimated or underestimated, depending on the baseline offset. The iPhone acceleration amplitude measurements were not affected by the changing weight distribution within the vehicle (Figure 7).

### Correlations of Acceleration Measures

**Figure 8:**
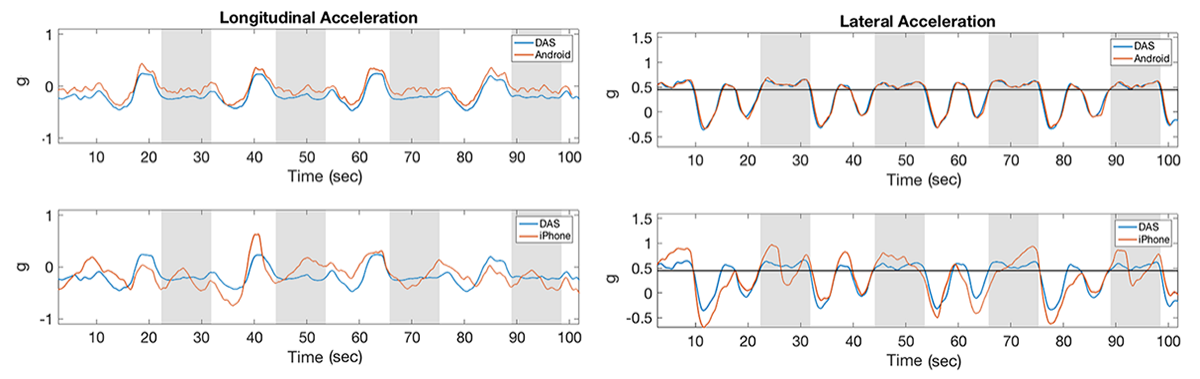
Acceleration measurements for hard left cornering maneuvers. The horizontal line along lateral acceleration represents the threshold of 0.45 g. Shaded stripes identify the approximate time of the cornering maneuvers. DAS: data acquisition system.

**Figure 9:**
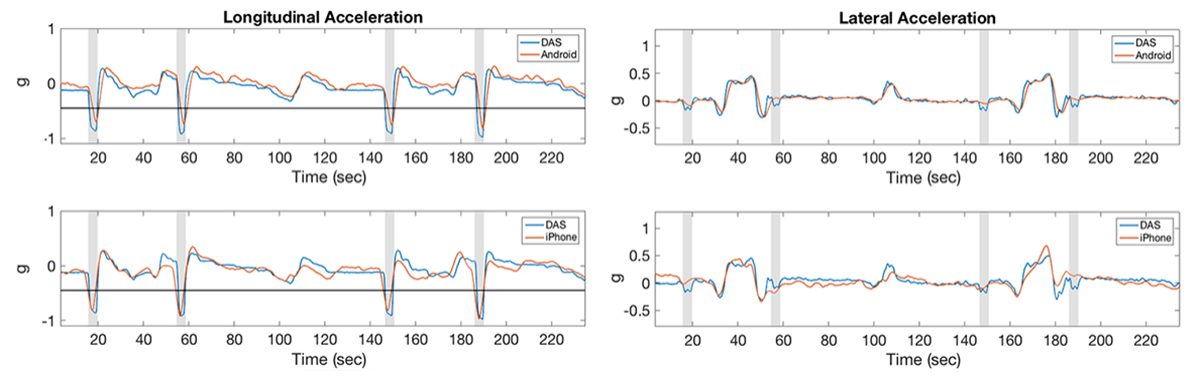
Acceleration measurements for hard braking maneuvers. The horizontal line along longitudinal acceleration represents the threshold of -0.45 g. Shaded stripes identify the approximate time of the braking maneuvers. DAS: data acquisition system.

**Figure 10:**
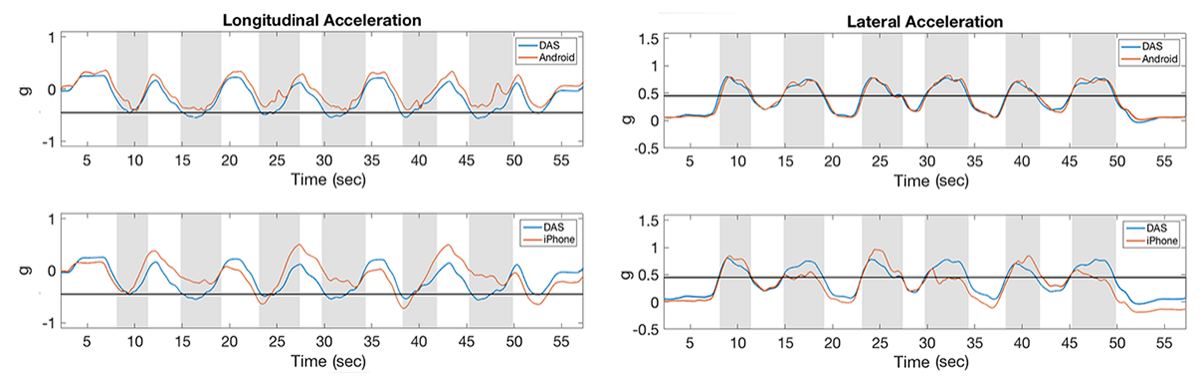
Acceleration measurements for hard left turning maneuvers. The horizontal line along longitudinal and lateral accelerations represents thresholds of –0.45 g and 0.45 g, respectively. Shaded stripes identify the approximate time of the turning maneuvers. DAS: data acquisition system.

In the test track assessment, the correlation coefficients between acceleration measurements acquired with DAS and Android devices were consistently higher than the correlation between DAS and iPhone devices for the same measurement, possibly because neither of these devices correct for gravity (Figures 8, 9, and 10). Averaging all maneuvers, the correlation coefficients between longitudinal and lateral accelerations between iPhone and DAS were *r*_lng_=0.71 and *r*_lat_=0.83, respectively, while the corresponding acceleration measurements between Android and DAS were *r*_lng_=0.95 and *r*_lat_=0.97 (Table 1). This study also revealed that the correlation coefficients between the iPhone and DAS lateral accelerations were higher than the corresponding longitudinal accelerations for most maneuvers (Table 1). A similar lateral versus longitudinal correlation difference was observed between the Android and DAS measurements (Table 1).

As shown in Table 2, the average duration of the g-force events above 0.45 g for the hard cornering maneuvers in the lateral direction were 9.5, 8.4, and 9.5 seconds for the DAS, iPhone, and Android devices, respectively. Similarly, the average duration of the hard-braking maneuvers above threshold were 3.5, 3.4, and 3.1 seconds for the DAS, iPhone, and Android devices, respectively. The average duration of the g-force events above 0.45 g during the hard turns were 3.9, 2.7, and 3.6 seconds for the DAS, iPhone, and Android devices, respectively.

**Figure 11:**
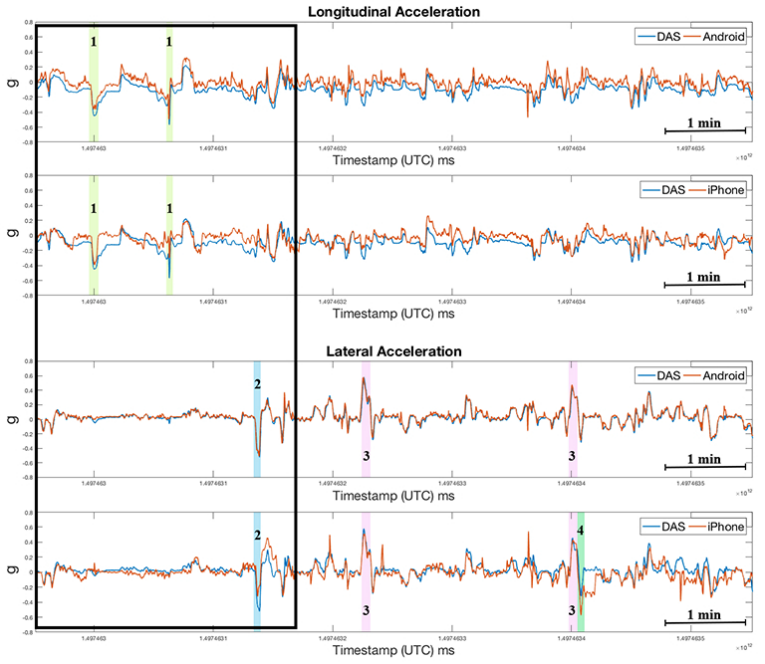
Acceleration measurements acquired during street driving in Christiansburg, VA. Highlighted are maneuvers that exceeded 0.45 g threshold: hard brake (1), hard right turn (2), hard left U-turn (3), and pothole (4). Details during first 3.4 minutes of driving within the black box are annotated in Figure 12. DAS: data acquisition system; UTC: Coordinated Universal Time.

**Figure 12:**
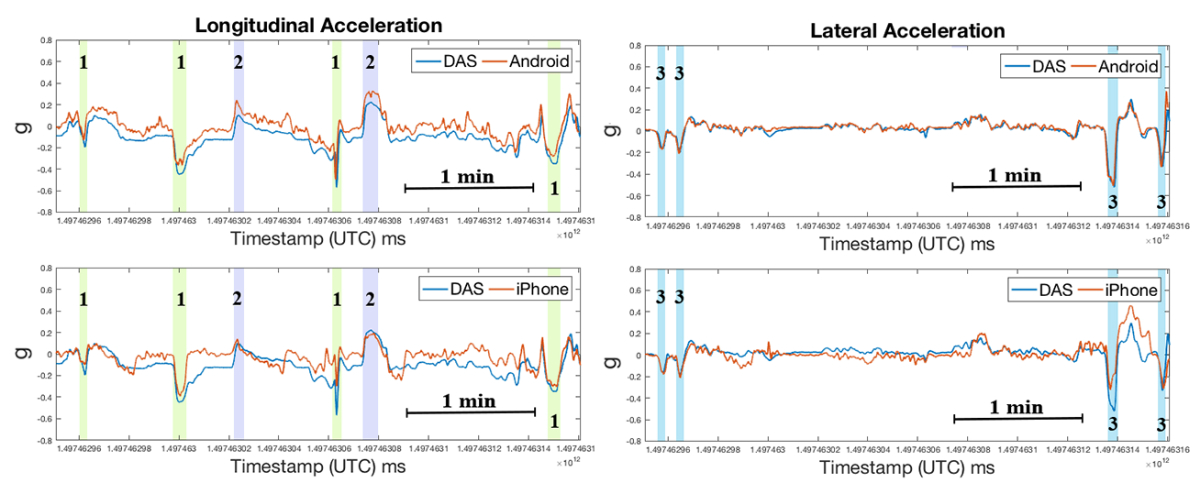
Acceleration measurements acquired during first 3.4 minutes of street driving in Christiansburg, VA (black box in Figure 11): brake (1), acceleration (2), and right turn (3). DAS: data acquisition system; UTC: Coordinated Universal Time.

In the street driving phase, the correlation coefficients (Figure 11 and Figure 12) were lower than those for the data obtained during experimental testing on the Smart Road, perhaps due to false positives (eg, speedbumps and potholes). The correlation coefficient between the iPhone and DAS longitudinal and lateral accelerations were *r*_lng_=0.62 and *r*_lat_=0.71, respectively; the corresponding correlation coefficients between the Android and DAS measurements where *r*_lng_=0.86 and *r*_lat_=0.91.

**Table 1:** Correlation coefficients between data acquisition system (DAS)/iPhone and DAS/Android devices.

Driving maneuver		*r*_lat_^a^		*r*_lng_^a^	
		iPhone	Android	iPhone	Android
**Cornering**					
	Left hard (>0.45 g)	0.86	0.94	0.65	0.88
	Left	0.82	0.99	0.58	0.93
	Right hard (>0.45 g)	0.78	0.97	0.63	0.98
	Right	0.80	0.99	0.77	0.96
Braking hard (>0.45 g)		0.76	0.93	0.84	0.89
**Turning**					
	Left hard (>0.45 g)	0.87	0.99	0.90	0.93
	Left	0.90	0.97	0.69	0.95
	Right hard (>0.45 g)	0.85	0.98	0.75	0.96
	Right	0.84	0.99	0.63	0.97
Average		0.83	0.97	0.71	0.95

^a^Pearson correlation coefficients, *r*, for acceleration along x (lateral, *r*_lat_) and z (longitudinal, *r*_lng_) axes.

**Table 2:** Average duration of the g-force events over 0.45 g threshold (seconds).

Driving maneuver		Lateral						Longitudinal				
**Cornering hard**												
	Left		9.8		8.0		9.9	0		0		0
	Right		9.2		8.8		9.1	0		0		0
Braking hard		0		0		0		3.5	3.4		3.1	
**Turning hard**												
	Left		4.2		3.1		3.8	2.0		2.1		0
	Right		3.6		2.2		3.4	0		0		0

## Discussion

### Principal Findings

The main objective of this study was to evaluate performance of a custom-built gForce iPhone app as a potential research tool. This evaluation compared the iPhone gForce acceleration measurements with data collected with the DAS device, which is the standard equipment in on-road NDSs. The findings indicated that correlations between measures of elevated g-forces from the iPhone gForce App and the DAS were reasonably high, ranging from r=0.78 to 0.81. This finding suggests that the iPhone 6 with integral sensor-fusion technology may be a viable data acquisition platform for detecting elevated g-forces, with the added benefit of decoupling measurements from gravity (ie, vehicle orientation relative to gravity). In comparison, Android and DAS measurements were highly sensitive not only to road topography (eg, road lateral slope or banking), but also to the weight distribution within the vehicle (ie, vehicle lean), thus altering acceleration amplitudes along individual axes. Due to this sensitivity, the average durations of the elevated g-force events captured with the iPhone device differed from the corresponding durations acquired with DAS and Android devices. This effect was especially pronounced during cornering and turning, since the vehicle was leaning during these maneuvers. In contrast, the highest similarity between the average durations of the elevated g-force events for all three devices was observed during braking, since this maneuver was less susceptible to vehicle leaning.

Future research will examine the association between g-forces collected by the iPhone and a device that includes in-built gravity adjustment, and is unaffected by weight distribution in the vehicle. Beyond this, the utility of the app would be assessed by recruiting participants to use the app while driving their own vehicles under usual driving. In this context, participant preferences for downloading an app on their existing iPhone device or using a dedicated device for data collection would need to be established. Given the sophistication of the iPhone and iOS development platform, the capabilities of the app could be extended to include video capture, real-time driver feedback, and cloud-based data aggregation and analyses. Finally, machine learning-based methods will be needed for automated maneuver identification and improved rejection of false positive g-force events not associated with a risky driving behavior.

### Conclusions

The gForce iPhone app reliably assessed elevated g-force events compared to the DAS. Collectively, the gForce app and iPhone platform have the potential to serve as feature-rich, inexpensive, scalable, and open-source tool for assessment of KRD events, with potential for research and feedback forms of intervention.

## Abbreviations

DAS: data acquisition system
DMP: digital motion processor
GPS: Global Positioning System
KRD: kinematic risky driving
NDS: naturalistic driving study
VTTI: Virginia Tech Transportation Institute
